# Effects on patient activation of eHealth support in addition to standard care in patients after radical prostatectomy: Analysis of secondary outcome from a randomized controlled trial

**DOI:** 10.1371/journal.pone.0308555

**Published:** 2024-09-10

**Authors:** Camilla Wennerberg, Mirjam Ekstedt, Kristina Schildmeijer, Amanda Hellström

**Affiliations:** 1 Department of Health and Caring Sciences, Linnaeus University, Kalmar, Sweden; 2 Department of Surgery, Region Kalmar County, Kalmar, Sweden; 3 Department of Learning, Management, Informatics and Ethics, Karolinska Institutet, Solna, Sweden; Almoosa College of Health Sciences, SAUDI ARABIA

## Abstract

**Introduction:**

Prostate cancer is often treated with radical prostatectomy, but surgery can leave patients with side effects. Patients who actively take part in their rehabilitation have been shown to achieve better clinical outcomes. eHealth support has the potential to increase patient activation, but has rarely been evaluated in long-term randomized controlled trials. Therefore, we evaluated the effects on patient activation of eHealth support (electronic Patient Activation in Treatment at Home, ePATH) based on motivational theory. The aim was to investigate the effects of eHealth support on patient activation at 6 and 12 months after radical prostatectomy, compared with standard care alone, and associations with baseline patient activation and depression.

**Methods:**

A multicentre randomized controlled trial with two study arms was conducted. Men planned for radical prostatectomy at three county hospitals in southern Sweden were included and randomized to the intervention or control group. The effects of ePATH on the secondary outcome, patient activation, were evaluated for one year after surgery using the patient activation measure and analysed using a linear mixed model.

**Results:**

The study included 170 men during 2018–2019. In the intervention group, 64% (53/83) used ePATH. The linear mixed model showed no significant differences between groups in patient activation [β -2.32, *P* .39; CI -7.64–3.00]. Baseline patient activation [β 0.65, *P* < .001; CI 0.40–0.91] and depression [β -0.86, *P* .03; CI -1.64– -0.07] statistically impacted patient activation scores over one year.

**Conclusions:**

ePATH had no impact on patient activation during long-term prostate cancer rehabilitation. However, patient activation at baseline and depression scores significantly influenced patient activation, underlining the need to assess these aspects in prostate cancer surgery rehabilitation.

**Trial registration:**

ISRCTN Registry ISRCTN18055968, (07/06/2018); https://www.isrctn.com/ISRCTN18055968; International Registered Report Identifier: RR2-10.2196/11625.

## Introduction

Prostate cancer is one of the most common malignancies in the male genitourinary system. The Global Cancer Statistical Report by WHO, shows that there were about 1 400 000 new cases of prostate cancer worldwide in 2020, accounting for over 7% of malignancies. The incidence rate ranking third highest after breast and lung cancer worldwide, and eighth in mortality rate [1]. Treatments for prostate cancer increase the chance of survival but can lead to side effects such as life-long urinary incontinence, affected bowel habits, pain, and sexual dysfunction, significantly impacting health-related quality of life [2]. Being diagnosed with cancer may also impact psychological well-being, and depression is more prevalent in patients with cancer than in the general population [3–5].

There is a growing awareness of the importance of patients having an active role in the management of their health and care, and the complex challenge of achieving that [6, 7]. Patient activation implies taking responsibility for managing symptoms, adhering to treatment regimens, and adopting new behaviours to reduce risks of complications [8]. However, standardized information routines often overlook assessing patients’ knowledge, skills and competence in actively managing their health and care [9–11].

Assessing patient activation informs strategies for supporting patients with varying capabilities in self-care. Patients range from being passive with poor adherence to recommendations to those who have changed their lifestyle, but struggle with maintaining these changes. With appropriate assessment and support, patients can improve their activation or maintain a high level [11, 12]. Patients with higher patient activation are more likely to understand their diagnosis, cope with side effects, adhere to treatment recommendations, adopt a healthy lifestyle, being satisfied with care [11, 13], and achieve more favourable clinical outcomes [13, 14]. To promote motivation and achieve high levels of patient activation, it is essential to satisfy three basic psychological needs: competence, autonomy, and relatedness [15]. While, adequate support can fulfil patient´s motivational needs, motivation is intricate. It varies on a continuum from intrinsic motivation (self-supporting inner will) to external factors driven by rewards or punishments [16]. Motivation is also vulnerable to external influences including persistent depression during prostate cancer rehabilitation. These influences pose additional challenges in health management [17, 18] affecting treatment decisions, adherence to recommendations, and functional outcomes [3].

Motivational interventions, rooted in fulfilling basic needs for intrinsic motivation have proven effective in cancer rehabilitation [19] and positively impact health outcomes [20]. eHealth interventions hold significant potential for promoting patient activation by offering integrated, individualized services that enhance accessibility, efficiency and align with patient preferences and location [20, 21]. However, there is a lack of longitudinal clinical randomized controlled trials (RCT), investigating the effects of eHealth interventions [22]. In a recent systematic review of eHealth interventions for patient activation, lectures and self-reports were found helpful in rehabilitation [21]. However, requests for interventions throughout the entire care trajectory (not just the initial phases) were noted [23]. Most eHealth interventions primarily focus on clinical outcomes, neglecting the potential to enhance patient activation or to consider its complexity [24]. This study sought to meet this gap by assessing the effects of an eHealth intervention on patient activation during prostate cancer rehabilitation.

The aim was to investigate the effects of eHealth support on patient activation at 6 and 12 months after radical prostatectomy, compared with standard care alone, and explore associations with baseline patient activation and depression.

## Methods

### Study design

This study analyses data from a RCT, with two study arms. It compares the effects of an eHealth support, known as *Patient Activation in Treatment at Home* (ePATH), which provides additional assistance for patients to adhere to rehabilitation recommendations in the intervention group, against standard care alone in the control group. The study design has been described in detail in the study protocol [25] and in a previous report on primary outcomes [26]. The study is registered, as an International Standard Randomized Controlled Trial (18055968), International Registered Report Identifier (10.2196/11625) and follows CONSORT guidelines (S1 Checklist).

### Ethics approval and consent to participate

The study was performed in accordance with principles in the Declaration of Helsinki and had ethical approval from the Regional Ethics Committee (Ref No 2016/484-31; 2017/512-32; 2018/147-32) in Sweden. All patients gave written informed consent to participate and were informed that data would be handled confidentially, and they could withdraw their consent at any time, without giving a reason.

### Inclusion criteria and sample size

The inclusion criteria were: >18 years, elective radical prostatectomy, having a smartphone-based e-identification, and being able to communicate in Swedish. All men who met the inclusion criteria and accepted participation at any of three county hospitals in southern Sweden were included. An exclusion criterion was having a cognitive impairment (assessed by the cancer nurse specialist).

An independent statistician performed the sample size estimation based on the “The sample size and suggested end point” table of the primary outcome measure for the RCT, the Expanded Prostate Index Composite (EPIC). To show clinical importance, the effect size was set to 0.5, as recommended. A maximum of 4 domains of EPIC, with a two-tailed independent samples t-test per domain, a 5% overall significance level and a power of 80%, 91 patients per group had to be included. Considering a dropout of 25%, 114 patients per group was needed [26–28]. Sample size was not calculated for the secondary outcome measure, the Patient Activation Measure (PAM), on which the present study reports [25].

### Setting and standard care

Three county hospitals in southern Sweden included patients planned for radical prostatectomy. The care organizations differed somewhat between the sites [25], but all clinics applied the national standardized care trajectory [9]. Men with prostate cancer are allocated a cancer nurse specialist, for easy access to healthcare and support. Generic recommendations for rehabilitation were provided verbally and in writing to both intervention and control groups. The cancer nurse specialists contacted the patients in conjunction with regular check-ups after surgery (usually at 3- or 6-month intervals) and provided individual consultation on rehabilitation and further treatment. Other contacts were on each patient’s initiative [9].

### The ePATH intervention

ePATH consisted of a web-based and mobile application, to be used as additional support in cancer rehabilitation. The intervention is based on the self-determination theory, to satisfy the three basic psychological needs for motivation: *competence*, *autonomy*, and *relatedness* [15]. *Competence* was supported as patients could read/re-read individualized information, explanations, and instruction for self-care at any time. Patient *autonomy* was respected as they had the option to customize ePATH, and independently choose actions and when to perform them. Patients could register self-care activities performed, rate their symptoms, get notifications, set goals for themselves and see graphs of improvements over time. *Relatedness* was pursued through the simplified interaction with the cancer nurse specialist through ePATH’s messaging function. Links to patient associations were also provided [29].

### Data collection and procedures

#### Recruitment

Patients were recruited between 2018 (Jan 1) and 2019 (Dec 31), for the study length to be reasonable and data collection was finalized in 2021 (Jan 31). In conjunction with treatment decisions on radical prostatectomy, all men at the three sites got written information about the study and a manual for ePATH from their cancer nurse specialist. The cancer nurse specialists asked the patients about participation a week after the written information. A signed consent form was sent by post to the researchers. Every other week, the cancer nurse specialists were in contact with CW regarding patient inclusion, at which time participants were sent the baseline survey. All participants answered web surveys at baseline (2 weeks prior to surgery) and at 1, 3, 6, and 12 months [25] after their radical prostatectomy (two reminders/survey were sent by e-mail within 10 days). This study reports on the patient activation and depression scores, reported at baseline, 6 and 12 months post radical prostatectomy.

#### Randomization

The trial was performed as a 1:1 randomization trial, with computer-generated block randomization lists created by an independent statistician. The allocation was revealed by opening sealed, numbered envelopes consecutively. ePATH accounts were set up (by CW) for the patients in the intervention group and the cancer nurse specialists adjusted the accounts based on each individual patient. All patients received an e-mail one week after the baseline questionnaires, revealing allocation. Thus, no blinding of patients, cancer specialist nurses or CW was possible. Both groups got standard care, and the intervention group additionally got access to ePATH for one year. They were free to use and adjust their accounts to suit individual preferences during this time.

### Measures

The characteristics of participants obtained at baseline were age, marital status, household income, and education. Use of ePATH was based on data metrics after data collection was finalized. Self-rated general health was measured using a single item from the Short Form Health Survey (SF-36) (in general, how would you describe your overall health?) rated on a 5-point scale from 1 = excellent to 5 = poor [30, 31].

Motivation was measured using the short form of the Needs Satisfaction and Frustration scale [32], with three subscales, a patient’s perceived autonomy, social belonging, and own competence. The scale comprises 6 items (2 per subscale), with 7 response options ranging from very often (1) to very seldom (7). One of two items per subscale should be reversed before summarizing and divided by two, for the final score. A Swedish version of the scale has been validated in a general Swedish population [33].

The Patient Health Questionnaire 9 (PHQ-9) comprises 9 questions, measuring the severity of depression (as defined by the Diagnostic and Statistical Manual of Mental Disorders 4^th^ edition), with a total score ranging from 0 to 27, and a cut-off score at 10. Higher scores indicate a higher degree of depression, with scores categorized on a five-level scale: minimal depression 1–4, mild depression 5–9, moderate depression 10–14, moderately severe depression 15–19, and severe depression 20–27. One item assesses suicide risk [34]. The PHQ-9 fulfils the criteria for sensitivity and specificity of instruments used to diagnose and grade depression severity [35]. The Swedish version has demonstrated good measurement properties in a primary care context and in a sample of psychiatric patients [36].

Patient activation was measured using the Patient Activation Measure (PAM-13), which aims to assess patient’s self-reported knowledge, skill, and confidence regarding management of own health and illness [12]. Patient activation is assessed by 13 items, that gauges a person’s self-concept as a manager of their own health and care. Each question has 5 response categories, ranging from strongly disagree (1) to strongly agree (4), with 0 corresponding to ‘not applicable’. A scoring sheet provided by the developer, Insignia Health, indicates the PAM scores (0–100) and correlating levels (1–4) of patient activation. Level 1: disengaged and overwhelmed; level 2: becoming aware, but still struggling; level 3: taking action; level 4: maintaining behaviours and pushing further. Each point change in the score is meaningful and a change of 3–4 points may be associated with the difference between being engaged and not. However, no cut-offs are available. Most individuals have activation scores between 30 and 90 [37]. The PAM-13 has shown good measurement properties regarding validity and reliability [12, 38] and the Swedish version has been validated in discharged medical patients, geriatric patients, and surgical patients [39].

### Statistical analysis

**Fig 1:**
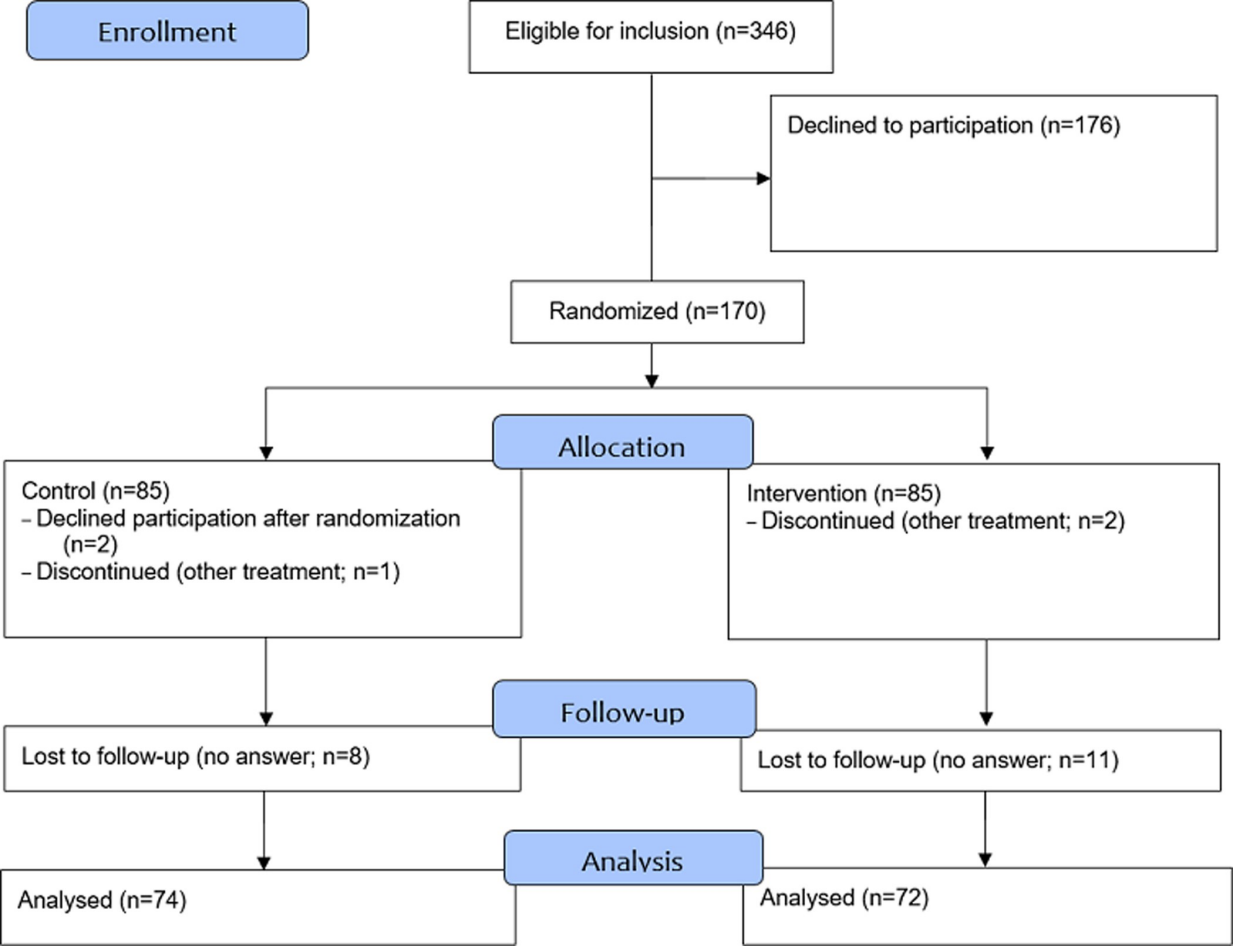
CONSORT flow diagram. 170 men were included and both groups allocated 85 men. Five men withdrew participation. Men with no answers (19) were considered lost to follow-up.

Data were analysed using SPSS software (version 29; IBM Corp) for Windows [40]. Of the 170 patients included, five declined participation after randomization (remaining: 83 intervention and 82 control group). Patients without any data (n = 20) were considered lost to follow-up and excluded from final analyses, resulting in 145 patients (71 intervention and 74 control group) included in a modified intention-to-treat analysis (Fig 1) [26]. For an intention-to-treat analysis to be valid, its assumptions need to be valid; not all patients must be included in the analysis [41]. A missing data analysis showed randomly missing data at the individual level for approximately 15% of outcome measures, no outliers were removed. We therefore imputed data, using multiple imputations with predictive means matching [42]. Five imputation rounds were performed, and the pooled values of these imputations have been presented [43]. The pooled values and imputations were further compared with the original data for validity (S1 Table). All statistical tests employed a two-sided significance level of < .05. Descriptive statistics were used to describe participant characteristics and baseline data. Means and standard deviations were used for the normally distributed continuous variables (S2 Table). To identify differences between groups, Pearson’s chi-squared test was used for nominal variables, the Mann-Whitney U-test for ordinal variables, and the independent Student’s t-test for normally distributed continuous variables. Some response options for self-rated general health were merged– 1 (poor) and 2 (fair), and 4 (very good) and 5 (excellent), respectively–due to a small number of responses at the extremes, resulting in a 3-point scale. To investigate the mean PAM score differences over time between groups, a linear mixed model with repeated measures and an unstructured correlation structure was used. An interaction term between time and group was employed to identify differences in the pattern of change. The assumptions of the model were checked based on residuals and Akaike Information Criterion (AIC). The model was controlled for the effects of baseline PAM scores, PHQ scores, and age. To illuminate the time-varying covariance of the PHQ, we used mean PHQ scores to distinguish from changes in time.

## Results

### Participant characteristics, baseline data, and use

The mean age of participants in both groups at inclusion was 64 years (SD 6.2) and age ranged from 48 to 78 years. The participant characteristics did not statistically significantly differ between the groups as regards marital status (p = .82), household income (p = .51), or level of education (p = .74). In the intervention group 43% (36/83) were married/had a partner compared to 54% (44/82) in the control group. The largest proportion of participants had a monthly household income >45,000 Swedish crowns (intervention group 40%, 33/83; control group 39%, 32/82), and university education (intervention group 27%, 22/83; control group 29%, 24/82).

Baseline data (Table 1) on the motivational subscales ‘autonomy’ and ‘relatedness’ and depression were similar between groups. However, the intervention group had higher PAM scores and higher ratings on the motivational subscale ‘competence’ than those in the control group at baseline. The largest proportion of participants (in both groups) had minimal depression at baseline. Use of ePATH in the intervention group was defined as using ePATH more than once, and 64% of the intervention group fell into that category.

**Table 1:** Baseline data (n = 165).

Characteristic	Intervention group n = 83	Control group n = 82
General health, n (%)		
Poor/Fair	9 (11)	5 (6)
Good	20 (24)	18 (22)
Very good/Excellent	30 (36)	41 (50)
Self-determination, mean (SD)		
Autonomy	2.33 (1.00)	2.26 (0.91)
Competence	2.63 (0.98)	2.25 (1.05)
Relatedness	2.18 (1.07)	1.97 (1.00)
Patient Health Questionnaire, n (%)		
Minimal	35 (42)	42 (51)
Mild	4 (5)	5 (6)
Moderate	2 (2)	1 (1)
Moderately severe	0 (0)	0 (0)
Severe	0 (0)	0 (0)
Patient activation scores, mean (SD)	71.56 (12.67)	67.02 (11.56)

SD: standard deviation.

### Changes in depression scores (PHQ-9)

The depression scores (PHQ-9 mean value) increased from 6 months to 12 months from minimal depression (mean 2.72 (SD 3.94)) to moderate depression (mean 12.92 (SD 4.11)) in both the intervention and control group. No statistically significant difference between groups was detected (6 months p = .69; 12 months p = .68).

### Effects of ePATH on patient activation (Patient Activation Measure, PAM-13)

The linear mixed model did not detect any statistically significant differences in PAM score between the intervention and control groups at 6 or 12 months (Table 2). No statistically significant interaction between time and group was found. Higher PAM score at baseline (higher patient activation) was statistically significantly correlated with increased PAM score at 6 and 12 months. Higher PHQ score (higher levels of depression) was statistically significantly correlated with decreased PAM score (less patient activation) at 6 and 12 months. Age did not statistically significantly correlate with PAM score.

**Table 2:** Changes in patient activation over time (6 and 12 months), linear mixed model.

	Estimate (β)	P value	CI 95%
Intercept	47.77	< .001	20.61 to 74.92
Intervention group vs. control group	-2.32	.39	-7.64 to 3.00
Time (12 months vs. 6 months)	-.09	.96	-3.35 to 3.53
Interaction: Group and time	-2.52	.31	-7.43 to 2.40
PAM baseline	.65	< .001	0.40 to 0.91
PHQ (mean over time)	-.86	.03	-1.64 to -0.07
Age	-.22	.19	-0.54 to 0.11

CI: confidence interval, PAM: Patient Activation Measure, PHQ: Patient Health Questionnaire.

Both intervention and control groups had improved PAM scores between 6 months and 12 months (Table 3).

**Table 3:** Mean values (PAM) at 6 and 12 months (linear mixed model).

	6 months		12 months	
	Mean value	CI 95%	Mean value	CI 95%
Intervention group	68.81	65.39–72.23	71.42	68.10–74.74
Control group	73.64	70.60–76.69	73.73	69.95–77.52

CI: confidence interval.

## Discussion

The present study investigated the effects of eHealth support on patient activation at 6 and 12 months after radical prostatectomy, compared with standard care alone, and explored associations with baseline patient activation and depression. Our results did not show an increase in patient activation in the intervention group although ePATH was designed based on self-determination theory [15]. Hence, ePATH did not effectively promote patients’ active engagement in long-term prostate cancer rehabilitation. Notably, both the intervention and control groups exhibited high motivation and patient activation at baseline. Although the intervention group had slightly higher scores in patient activation and competence than the control group, it is possible that patients with already fulfilled motivation needs may not experience further improvements. High PAM scores could indicate that these patients possess the necessary skills and confidence to seek rendering additional support targeting motivational aspects unnecessary [13].

Our study shows that patients’ PAM scores at baseline significantly correlated with PAM scores at 6 and 12 months. Patients in both groups had high PAM scores at baseline and these were maintained over the year after surgery. Previous research indicates that when a behaviour is established at baseline, increase in patient activation correlates with sustained high levels of that behaviour over time [44]. However, ePATH, which broadly supports patient activation, did not differentiate interventions for highly activated versus less activated patients. Assessing patient activation at the beginning of a patient’s cancer trajectory can help identify those who require targeted support [45]. Healthcare professionals could need tools for tailoring support based on each patient’s capacity for activation [46].

The streamlined accessibility to the cancer nurse specialist in ePATH did not yield an impact on patient activation despite the possibility of increased engagement. Prior research [47], underscores that substantial interaction time with a care provider correlates with increased activation levels among prostate cancer patients. This suggests that strategies aimed at enhancing accessibility to healthcare professionals could be a promising approach for fostering patient activation. Nevertheless, our results did not support ePATH as such a tool. As the demand for cancer rehabilitation support grows and care structure evolves [1], enhancing patient activation might require re-evaluating the roles of patients and healthcare providers, involving both parties in utilizing eHealth tools, as their interaction significantly influences outcomes.

Depression (PHQ scores) negatively predicted patient activation, impacting self-management and adherence to rehabilitation recommendations, which is in line with previous research [48]. Prior research found that patients with higher patient activation correlated with lower depression scores over one year and that more activated patients showed greater engagement in healthy behaviours [49]. Further, psychological interventions in prostate cancer rehabilitation also improved depression scores [50–52]. Thus, to effectively address patient needs, supporting patient activation may require integrating the management of depression symptoms [49]. However, further research is needed to explore causal relationships and effective interventions for behaviour change and increased patient activation [5].

eHealth has the potential to improve patient activation through interventions [53] but is rarely incorporated into the healthcare system [54]. Patients are left to decide on their own, which applications might be useful for them. There is a need for further understanding of the mechanisms determining what works, under which circumstances, and for whom, to disseminate eHealth interventions in cancer rehabilitation [55, 56]. Future research should aim to assess not only the outcomes of eHealth interventions on patient activation, but also explore the specific mechanisms through which eHealth support can be beneficial. It is crucial to investigate which patient groups can benefit from eHealth interventions.

### Strengths and limitations

The study’s power was calculated based on primary outcome measures in another study [26]. Recruitment of patients was terminated after two years, of concern of the involved clinics (workload) and for standard care to remain the same for all included participants. The current study therefore included 170 patients. Dropout can be a threat to validity, as it can introduce selection bias. An intention-to-treat analysis, where dropouts are assumed to have a neutral effect, is a way to avoid bias. However, such a procedure can diminish the power of a study [57]. eHealth interventions generally struggle with high dropout rates and one of the fundamental methodological issues in eHealth trials is that a substantial proportion of the intervention group will use the intervention sparingly or not at all [58]. The study was performed in routine clinical practice, to ensure minimal burden on the valuable time of the cancer nurse specialists and to show how this kind of intervention could work in contemporary clinical scenarios. This approach stems from the prevalent demand for clinical RCTs [22]. The frequency of use of ePATH [26] in the intervention group might have affected the results, as not all men used ePATH, even though they had gone through recruitment and given informed consent, thus agreeing to adopt the intervention [57]. Participants might have experienced dissonance between the intervention and their expectations, affecting attrition [59] or not perceived as creating benefits [57]. The procedure at inclusion might have influenced the use as well; incorporation of a hands-on introduction or choosing another time point might have increased use. Multiple factors affect how the findings can be generalized and eHealth support do not reach the total population. Further studies should focus on investigating which patients would gain from eHealth support for patient activation.

Applicability and validity should be considered in the evaluation. The study had a homogeneous sample of men after radical prostatectomy, which increases the generalizability of the findings. The sample had high PAM scores in general, which reflects the physiological capacity of this population. Further, that fact raises questions of selection bias and ceiling effects, causing difficulties in identifying statistical differences between groups. The use of linear mixed models with two assessment points improved the representability of the sample, as individuals without complete data could be included [60]. However, the use of imputations should be considered. An accepted method for imputation was used, which increases validity [61]. Valid results were seen when the imputed dataset was compared with the original dataset.

Patient activation is a complex outcome with heterogeneous variables and processes involved. The lack of conceptual clarity concerning patient empowerment, engagement, and activation, makes it challenging to operationalize interventions and measure all the aspects involved, influencing the eHealth context [62]. Although it appears that eHealth interventions can be effective, the interventions show a great heterogeneity of technologies, variables, and outcomes assessed [20]. The PAM offers an adequate measure to monitor patient activation [12] and is viewed as a successful way to measure patient activation [63], but can be arbitrary in nature [64]. In the present study, patients with elevated depression scores correspondingly showed diminished patient activation scores. This underscores the importance of illuminating the complex dimension of patient activation within the context of cancer rehabilitation. Moreover, it emphasises the need for a comprehensive approach where the evaluation and support of patients encompass both depression and patient activation in parallel throughout the rehabilitation process.

## Conclusions

ePATH had no impact on patient activation during long-term prostate cancer rehabilitation. However, baseline patient activation levels and depression scores significantly influenced patient activation. This study underlines the need to assess patient activation preoperatively and identify depression in post-prostate cancer surgery rehabilitation.

## Supporting information

S1 Checklist CONSORT checklist. (DOC)

S1 Table Sensitivity analysis of original data and five imputation rounds. (XLSX)

S2 Table Descriptive statistics of mean (SD) and median (IQR) of continuous outcome variables. (DOCX)

## List of abbreviations

ePATH: electronic Patient Activation in Treatment at Home
EPIC: Expanded Prostate cancer Index Composite
RCT: Randomized Control Trial
PAM: Patient Activation Measure
SF: Short Form Health Survey
PHQ: Patient Health Questionnaire
SD: Standard Deviation
SEK: Swedish Crown
CI: Confidence Interval

## References

[1] Sung H, Ferlay J, Siegel RL, Laversanne M, Soerjomataram I, Jemal A, et al. Global cancer statistics 2020: GLOBOCAN estimates of incidence and mortality worldwide for 36 cancers in 185 countries. CA Cancer J Clin. 2021;71(3):209–249. doi: 10.3322/caac.21660 33538338

[2] Moris L, Cumberbatch MG, Van den Broeck T, Gandaglia G, Fossati N, Kelly B, et al. Benefits and risks of primary treatments for high-risk localized and locally advanced prostate cancer: An international multidisciplinary systematic review. Eur. Urol. 2020;77(5):614–27. doi: 10.1016/j.eururo.2020.01.033 32146018

[3] Brunckhorst O, Hashemi S, Martin A, George G, Van Hemelrijck M, Dasgupta P, et al. Depression, anxiety, and suicidality in patients with prostate cancer: A systematic review and meta-analysis of observational studies. Prostate Cancer Prostatic Dis. 2021;24(2):281–9. doi: 10.1038/s41391-020-00286-0 32978524

[4] Friberg AS, Oksbjerg Dalton S, Benzon Larsen S, Andersen EW, Kroyer A, Helgstrand JT, et al. Risk of Depression After Radical Prostatectomy- A Nationwide Registry-based Study. Eur Urol Oncol. 2021;4(4):601–608. doi: 10.1016/j.euo.2019.06.020 31345731

[5] Crump C, Stattin P, Brooks JD, Sundqvist J, Bill-Axelson A, Edwards AC, et al. Long-term Risk of Depression and Suicide Among Men with Prostate Cancer: A National Cohort Study. Eur Urol. 2023;84(3):263–272) 37169640 10.1016/j.eururo.2023.04.026 PMC10523908

[6] Barello S, Graffigna G, Savarese M, Bosio AC. Engaging patients in health management: Towards a preliminary theoretical conceptualization. Psicol. Salute. 2014:11–33.

[7] Greene J, Hibbard JH. Why does patient activation matter? An examination of the relationships between patient activation and health-related outcomes. J. Gen. Intern. Med. 2012;27(5):520–6. doi: 10.1007/s11606-011-1931-2 22127797 PMC3326094

[8] Stout NL, Santa Mina D, Lyons KD, Robb K, Silver JK. A systematic review of rehabilitation and exercise recommendations in oncology guidelines. CA: Cancer J. Clin. 2021;71(2):149–75. 10.3322/caac.21639 PMC7988887 33107982

[9] Bratt O, Carlsson S, Fransson P, Thellenberg Karlsson C, Stranne J, Kindblom J. The Swedish national guidelines on prostate cancer, part 1: Early detection, diagnostics, staging, patient support and primary management of non-metastatic disease. Scand. J. Urol. 2022;56(4):265–73. doi: 10.1080/21681805.2022.2094462 35811480

[10] Frosch DL, Elwyn G. I believe, therefore I do. J. Gen. Intern. Med. 2011; 24:2–4. doi: 10.1007/s11606-010-1560-1 21061083 PMC3024097

[11] Hibbard JH, Mahoney E, Sonet E. Does patient activation level affect the cancer patient journey? Patient Educ Couns. 2017;100(7):1276–9. doi: 10.1016/j.pec.2017.03.019 28330715

[12] Hibbard JH, Stockard J, Mahoney ER, Tusler M. Development of the Patient Activation Measure (PAM): Conceptualizing and measuring activation in patients and consumers. Health Serv. Res. 2004;39(4p1):1005–26. doi: 10.1111/j.1475-6773.2004.00269.x 15230939 PMC1361049

[13] Westman B, Bergkvist K, Karlsson Rosenblad A, Sharp L, Bergenmar M. Patients with low activation level report limited possibilities to participate in cancer care. Health Expect. 2022;25(3):914–24. doi: 10.1111/hex.13438 35049103 PMC9122461

[14] Anderson G, Rega ML, Casasanta D, Graffigna G, Damiani G, Barello S. The association between patient activation and healthcare resources utilization: A systematic review and meta-analysis. Public Health. 2022;210:134–41. doi: 10.1016/j.puhe.2022.06.021 35970015

[15] Ryan, Deci E. Self-Determination Theory: Basic psycological needs in motivation, development and wellness. New York, London England: The Guilford Press; 2017.

[16] Vansteenkiste M, Ryan RM, Soenens B. Basic psychological need theory: Advancements, critical themes, and future directions. Motiv. Emot. 2020;44:1–31.

[17] Ahmed E. Antidepressants in patients with advanced cancer: When they’re warranted and how to choose therapy. Oncol. 2019;33(2):62–8. 30784031

[18] Naser AY, Hameed AN, Mustafa N, Alwafi H, Dahmash EZ, Alyami HS, et al. Depression and anxiety in patients with cancer: A cross-sectional study. Front. Psychol. 2021;12:585534. doi: 10.3389/fpsyg.2021.585534 33935849 PMC8081978

[19] Bonetti L, Tolotti A, Anderson G, Nania T, Vignaduzzo C, Sari D, et al. Nursing interventions to promote patient engagement in cancer care: A systematic review. Int. J. Nurs. Stud. 2022;133:104289. doi: 10.1016/j.ijnurstu.2022.104289 35751947

[20] Barello S, Triberti S, Graffigna G, Libreri C, Serino S, Hibbard J, et al. eHealth for patient engagement: A systematic review. Front. Psychol. 2016;6:2013. doi: 10.3389/fpsyg.2015.02013 26779108 PMC4705444

[21] Sánchez-Gutiérrez C, Gil-García E, Rivera-Sequeiros A, López-Millán JM. Effectiveness of telemedicine psychoeducational interventions for adults with non-oncological chronic disease: A systematic review. J. Adv. Nurs. 2022;78(5):1267–80. doi: 10.1111/jan.15151 35075690

[22] Camara Gradim LC, Archanjo Jose M, Marinho Cezar da Cruz D, de Deus Lopes R. IoT services and applications in rehabilitation: An interdisciplinary and meta-analysis review. IEEE Trans Neural Syst Rehabil Eng. 2020;28(9):2043–52. doi: 10.1109/TNSRE.2020.3005616 32746308

[23] Bader M, Zheng L, Rao D, Shiyanbola O, Myers L, Davis T, et al. Towards a more patient-centered clinical trial process: A systematic review of interventions incorporating health literacy best practices. Contemp Clin Trials. 2022;116:106733. doi: 10.1016/j.cct.2022.106733 35301134 PMC9196949

[24] Kelders SM, van Zyl LE, Ludden GDS. The concept and components of engagement in different domains applied to eHealth: A systematic scoping review. Front. Psychol. 2020;11:926. doi: 10.3389/fpsyg.2020.00926 32536888 PMC7266981

[25] Ekstedt M, Schildmeijer K, Wennerberg C, Nilsson L, Wannheden C, Hellstrom A. Enhanced patient activation in cancer care transitions: Protocol for a randomized controlled trial of a tailored electronic health intervention for men with prostate cancer. J. Med. Internet Res. Protoc. 2019;8(3):e11625. doi: 10.2196/11625 30900999 PMC6450475

[26] Wennerberg C, Hellström A, Schildmeijer K, Ekstedt M. Effects of Web-Based and Mobile Self-Care Support in Addition to Standard Care in Patients After Radical Prostatectomy: Randomized Controlled Trial. JMIR Cancer. 2023;9:e44320. doi: 10.2196/44320 37672332 PMC10512115

[27] Wei JT, Dunn RL, Litwin MS, Sandler HM, Sanda MG. Development and validation of the expanded prostate cancer index composite (EPIC) for comprehensive assessment of health-related quality of life in men with prostate cancer. Urol. 2000;56(6):899–905. doi: 10.1016/s0090-4295(00)00858-x 11113727

[28] Skolarus TA, Dunn RL, Sanda MG, Chang P, Greenfield TK, Litwin MS, et al. Minimally important difference for the expanded prostate cancer index composite short form. Urol. 2015;85(1):101–6. doi: 10.1016/j.urology.2014.08.044 25530370 PMC4274392

[29] Ekstedt M, Kirsebom M, Lindqvist G, Kneck Å, Frykholm O, Flink M, et al. Design and development of an eHealth service for collaborative self-management among older adults with chronic diseases: A theory-driven user-centered approach. Int. J. Environ. Res. Public Health. 2022;19(1):391. 10.3390/ijerph19010391 PMC8744716 35010652

[30] Hays RD, Morales LS. The RAND-36 measure of health-related quality of life. Ann Med. 2001;33(5):350–7. doi: 10.3109/07853890109002089 11491194

[31] Ohlsson-Nevo E, Hiyoshi A, Norén P, Möller M, Karlsson J. The Swedish RAND-36: Psychometric characteristics and reference data from the Mid-Swed Health Survey. J. Patient-Rep. Outcomes. 2021;5(1):1–11. 34347192 10.1186/s41687-021-00331-z PMC8339183

[32] Longo Y, Gunz A, Curtis GJ, Farsides T. Measuring need satisfaction and frustration in educational and work contexts: The Need Satisfaction and Frustration Scale (NSFS). J. Happiness Stud. 2016;17(1):295–317.

[33] Aurell J, Wilsson L, Bergström A, Ohlsson J, Martinsson J, Gustavsson P. Evaluation of the Swedish version of The Need Satisfaction and Frustration Scale (NSFS). Göteborg: Göteborg University. 2015.

[34] Kroenke K, Spitzer R, Williams J. The PHQ-9: Validity of a brief depression severity measure. Gen Intern Med. 2001; 16: 606–13. doi: 10.1046/j.1525-1497.2001.016009606.x 11556941 PMC1495268

[35] Pettersson A, Boström KB, Gustavsson P, Ekselius L. Which instruments to support diagnosis of depression have sufficient accuracy? A systematic review. Nord. J. Psychiatry. 2015;69(7):497–508. doi: 10.3109/08039488.2015.1008568 25736983

[36] Hansson M, Chotai J, Nordstöm A, Bodlund O. Comparison of two self-rating scales to detect depression: HADS and PHQ-9. Br. J. Gen. Pract. 2009;59(566):e283–e8. doi: 10.3399/bjgp09X454070 19761655 PMC2734374

[37] InsignaHealth. Research Patient Activation Measure Phreesia. URL: https://www.insigniahealth.com/research/research-licenses. [accessed 2021-01-10].

[38] Hibbard JH, Mahoney ER, Stockard J, Tusler M. Development and testing of a short form of the patient activation measure. Health Serv. Res. 2005;40(6p1):1918–30. 16336556 10.1111/j.1475-6773.2005.00438.x PMC1361231

[39] Hellström A, Kassaye Tessma M, Flink M, Dahlgren A, Schildmeijer K, Ekstedt M. Validation of the patient activation measure in patients at discharge from hospitals and at distance from hospital care in Sweden. BMC Public Health. 2019;19(1):1701. doi: 10.1186/s12889-019-8025-1 31856796 PMC6921492

[40] IBM SPSS statistics for Windows. IBM Corp. 2019. URL: https://www.ibm.com/products/spss-statistics [accessed 2022-08-30].

[41] Gupta SK. Intention-to-treat concept: a review. Perspect. Clin. Res. 2011;2(3):109. doi: 10.4103/2229-3485.83221 21897887 PMC3159210

[42] Marbach M. Choosing imputation models. Political Anal. 2022;30(4):597–605.

[43] Elliott P, Hawthorne G. Imputing missing repeated measures data: How should we proceed? Aust. N Z J Psychiatry. 2005;39(7):575–82. doi: 10.1080/j.1440-1614.2005.01629.x 15996138

[44] Hibbard JH, Mahoney ER, Stock R, Tusler M. Do increases in patient activation result in improved self‐management behaviors? Health Serv. Res. 2007;42(4):1443–63. doi: 10.1111/j.1475-6773.2006.00669.x 17610432 PMC1955271

[45] Nilsson L, Hellström A, Wennerberg C, Ekstedt M, Schildmeijer K. Patients’ experiences of using an e-Health tool for self-management support after prostate cancer surgery: a deductive interview study explained through the FITT framework. BMJ Open. 2020;10(6):e035024. doi: 10.1136/bmjopen-2019-035024 32601113 PMC7328745

[46] Vitger T, Korsbek L, Austin SF, Petersen L, Nordentoft M, Hjorthøj C. Digital shared decision-making interventions in mental healthcare: A systematic review and meta-analysis. Front. Psychiatry. 2021;12:691251. doi: 10.3389/fpsyt.2021.691251 34552514 PMC8450495

[47] O’Malley D, Dewan AA, Ohman‐Strickland PA, Gundersen DA, Miller SM, Hudson SV. Determinants of patient activation in a community sample of breast and prostate cancer survivors. Psycho‐Oncol. 2018;27(1):132–40. 10.1002/pon.4387 PMC5568503 28133892

[48] Magnezi R, Glasser S, Shalev H, Sheiber A, Reuveni H. Patient activation, depression and quality of life. Patient Educ. Couns. 2014;94(3):432–7. doi: 10.1016/j.pec.2013.10.015 24331277

[49] Sacks RM, Greene J, Hibbard JH, Overton V. How well do patient activation scores predict depression outcomes one year later? J. Affect. Disord. 2014;169:1–6. doi: 10.1016/j.jad.2014.07.030 25128858

[50] Mundle R, Afenya E, Agarwal N. The effectiveness of psychological intervention for depression, anxiety, and distress in prostate cancer: A systematic review of literature. Prostate Cancer Prostatic Dis. 2021;24(3):674–87. doi: 10.1038/s41391-021-00342-3 33750905

[51] Qan’ir Y, Song L. Systematic review of technology-based interventions to improve anxiety, depression, and health-related quality of life among patients with prostate cancer. Psycho-Oncol. 2019;28(8):1601–13. doi: 10.1002/pon.5158 31222956 PMC7465427

[52] Stein AT, Carl E, Cuijpers P, Karyotaki E, Smits JA. Looking beyond depression: A meta-analysis of the effect of behavioral activation on depression, anxiety, and activation. Psychol. Med. 2021;51(9):1491–504. doi: 10.1017/S0033291720000239 32138802

[53] Matamala-Gomez M, Maisto M, Montana JI, Mavrodiev PA, Baglio F, Rossetto F, et al. The role of engagement in teleneurorehabilitation: A Systematic Review. Front Neurol. 2020;11:354. doi: 10.3389/fneur.2020.00354 32435227 PMC7218051

[54] Latorre GFS, de Fraga R, Seleme MR, Mueller CV, Berghmans B. An ideal e-health system for pelvic floor muscle training adherence: Systematic review. Neurourol Urodyn. 2019;38(1):63–80. doi: 10.1002/nau.23835 30375056

[55] Escriva Boulley G, Leroy T, Bernetière C, Paquienseguy F, Desfriches-Doria O, Préau M. Digital health interventions to help living with cancer: A systematic review of participants’ engagement and psychosocial effects. Psycho-Oncol. 2018;27(12):2677–86. 10.1002/pon.4867 30152074

[56] Chiu TM, Eysenbach G. Stages of use: consideration, initiation, utilization, and outcomes of an internet-mediated intervention. BMC Med. Inform. Decis. Mak. 2010;10(1):1–11. doi: 10.1186/1472-6947-10-73 21092275 PMC3000372

[57] Eysenbach G. The law of attrition. J. Med. Internet Res. 2005;7(1):e402. doi: 10.2196/jmir.7.1.e11 15829473 PMC1550631

[58] Eysenbach G. Issues in evaluating health websites in an internet-based randomized controlled trial. J. Med. Internet Res. 2002;4(3):e867. 10.2196/jmir.4.3.e17 PMC1761938 12554548

[59] Rogers EM. Diffusion of innovations. Simon and Schuster; 2010.

[60] Fitzmaurice GM, Laird NM, Ware JH. Applied longitudinal analysis. John Wiley & Sons; 2012.

[61] Huque MH, Moreno-Betancur M, Quartagno M, Simpson JA, Carlin JB, Lee KJ. Multiple imputation methods for handling incomplete longitudinal and clustered data where the target analysis is a linear mixed effects model. Biom J. 2020;62(2):444–66. doi: 10.1002/bimj.201900051 31919921 PMC7614826

[62] Risling T, Martinez J, Young J, Thorp-Froslie N. Evaluating patient empowerment in association with eHealth technology: Scoping review. J. Med. Internet Res. 2017;19(9):e329. doi: 10.2196/jmir.7809 28963090 PMC5640823

[63] Newland P, Lorenz R, Oliver BJ. Patient activation in adults with chronic conditions: A systematic review. J. Health Psychol. 2021;26(1):103–14. doi: 10.1177/1359105320947790 32830587

[64] Graffigna G, Barello S, Bonanomi A, Lozza E. Measuring patient engagement: development and psychometric properties of the Patient Health Engagement (PHE) Scale. Front. Psychol. 2015;6:274. doi: 10.3389/fpsyg.2015.00274 25870566 PMC4376060

