# Supplementary material for: Effects on patient activation of eHealth support in addition to standard care in patients after radical prostatectomy: Analysis of secondary outcome from a randomized controlled trial
Source: PLoS One. 2024 Sep 10;19(9):e0308555. doi: 10.1371/journal.pone.0308555 (PMC11386445; doi:10.1371/journal.pone.0308555)
Supplement: S2 Table — (DOCX) [file pone.0308555.s003.docx]

| Supplementary file 3.  Descriptive statistics of mean (SD) and median (IQR) of continuous outcome variables. | | | |
| --- | --- | --- | --- |
|  |  | PAM | PHQ |
| Baseline | Mean | 69.12 | 2.15 |
|  | Median | 67.80 | 1.00 |
|  | SD | 12.25 | 2.70 |
|  | IQR | 14.40 | 3.00 |
|  |  |  |  |
| 6months |  |  |  |
|  | Mean | 71.56 | 2.72 |
|  | Median | 70.20 | 1.00 |
|  | SD | 16.17 | 3.94 |
|  | IQR | 20.3 | 3.00 |
|  |  |  |  |
| 12 months |  |  |  |
|  | Mean | 73.43 | 12.92 |
|  | Median | 71.35 | 11.00 |
|  | SD | 16.27 | 4.11 |
|  | IQR | 21.70 | 4.00 |
| PAM: Patient Activation Measure  PHQ: Patient Health Questionnaire | | | |
